# HER2 Expression in Bladder Cancer: A Focused View on Its Diagnostic, Prognostic, and Predictive Role

**DOI:** 10.3390/ijms24043720

**Published:** 2023-02-13

**Authors:** Francesca Sanguedolce, Magda Zanelli, Andrea Palicelli, Alessandra Bisagni, Maurizio Zizzo, Stefano Ascani, Maria Carmela Pedicillo, Angelo Cormio, Ugo Giovanni Falagario, Giuseppe Carrieri, Luigi Cormio

**Affiliations:** 1 Pathology Unit, Policlinico Riuniti, University of Foggia, 71122 Foggia, Italy; 2 Pathology Unit, Azienda USL-IRCCS di Reggio Emilia, 42123 Reggio Emilia, Italy; 3 Surgical Oncology Unit, Azienda USL-IRCCS di Reggio Emilia, 42123 Reggio Emilia, Italy; 4 Pathology Unit, Azienda Ospedaliera Santa Maria di Terni, University of Perugia, 05100 Terni, Italy; 5 Urology Unit, Azienda Ospedaliero-Universitaria Ospedali Riuniti Di Ancona, Università Politecnica Delle Marche, 60126 Ancona, Italy; 6 Department of Urology and Renal Transplantation, Policlinico Riuniti, University of Foggia, 71122 Foggia, Italy; 7 Department of Urology, Bonomo Teaching Hospital, 76123 Andria, Italy

**Keywords:** bladder cancer, HER2, immunohistochemistry, diagnosis, prognosis, therapeutic target

## Abstract

Bladder cancer (BC) is a heterogeneous disease from a molecular, morphological, and clinical standpoint. HER2 is a known oncogene involved in bladder carcinogenesis. Assessing HER2 overexpression as a result of its molecular changes in a routine pathology practice using immunohistochemistry might be a useful adjunct in several scenarios, namely (1) to correctly identify flat urothelial lesions and inverted urothelial lesions in the diagnostic setting; (2) to provide prognostic hints in both non-muscle invasive (NMI) and muscle invasive (MI) tumors, thus supplementing risk stratification tools, especially when evaluating higher-risk tumors such as those with variant morphology; (3) to improve antibody panels as a surrogate marker of BC molecular subtyping. Furthermore, the potential of HER2 as a therapeutic target has been only partly explored so far, in light of the ongoing development of novel target therapies.

## 1. Introduction

Bladder cancer (BC) is the seventh most prevalent malignancy and the thirteenth cause of cancer death worldwide, accounting for 1,720,625 cases overall and 573,278 newly diagnosed cases each year [1]. Approximately a quarter of BC patients present with advanced (muscle-invasive or metastatic) disease (MIBC), whereas the remaining 75% are diagnosed with non-muscle invasive BC (NMIBC), with both having different clinical behavior and therapeutic strategies [2,3]. TURBT/radiotherapy/chemotherapy is not comparable to radical cystectomy, though responses to treatment may be variable and sometimes barely predictable. Novel target treatment options have been introduced in the advanced setting of the disease, including anti-Fibroblast Growth Factor Receptor (FGFR) and immune checkpoint inhibitors (ICIs), as well as antibody–drug conjugates (ADCs) (see below) [4]. 

On the other hand, most NMIBC patients undergo favorable outcomes upon early diagnosis; nevertheless, these tumors carry both high recurrence rates and a significant risk of progression to muscle-invasive disease, especially in high-risk cases, with inherent increased lifetime costs per patient [5]. Such high-risk tumors may be cured with transurethral resection along with intravesical instillations of chemotherapeutic or immunotherapeutic agents (namely, mitomycin C and Bacillus Calmette–Guérin (BCG)), or early cystectomy. Yet, the latter may carry postoperative life-changing disadvantages, whereas BCG intravesical therapy may be ineffective and yield a high rate of adverse effects, with a varying potential to disease relapse and progression [2,3]. 

The underlying biology of such clinical heterogeneity is still a matter of study. As far as we know, BC is a heterogeneous disease from a molecular standpoint as well, resulting from a complex multi-step carcinogenesis that includes several changes involving genes and molecular pathways with specific functions in tumor development and progression. In an attempt to catch this biological variability, several efforts have been made over the last decade to develop of a molecular classification encompassing a discrete category of BC harboring different clinical and prognostic features, matching their DNA and RNA profiles [6,7,8,9]. In routine practice, current risk assessment models in NMIBC, as well as predictive/prognostic systems in MIBC, are based on clinical and histopathological features [10]. During the last few years, attempts have been made to improve the management of BC patients by introducing more effective risk stratification tools, including molecular markers; as a result of this, a more comprehensive analysis of genomic, epigenetic, and transcriptomic features have been accomplished, providing novel insights into bladder carcinogenesis [9,11]. The ultimate goal is to find biomarkers with a prognostic/predictive role and which, hopefully, may act as potential therapeutic targets as well; they should be based upon a clear rationale, detectable both in vitro and in vivo in distinct specimens (urine, blood, tissue) using selected technologies, and be easily assessed and quantified [12]. Finally, they should demonstrate clinical soundness and utility upon assay standardization, threshold establishment, and external validation [13].

Human Epidermal growth factor Receptor 2 (HER2/ERBB2) is a member of a family of epithelial growth factor receptors, along with HER1/EGFR, HER3, and HER4 [14], which are transmembrane receptor tyrosine kinases involved in cell proliferation, survival, and mobility via the downstream activation of different intracellular signaling pathways such as the mitogen-activated protein kinase (MAPK) and the phosphoinositide 3-kinase (PI3K/Akt) pathways [14]. The HER2 proto-oncogene, located at the long arm of chromosome 17 (17q12), codes for the HER2 protein, and its mutation and amplification mostly result in HER2 overexpression [14]. Higher HER2 levels have well-known prognostic and predictive roles in breast and gastroesophageal cancers, where immunohistochemistry (IHC) is routinely used to assess HER2 status at the protein level [15]. In this setting, patients are stratified according to the presence/absence, intensity, and completeness of the membrane staining in the tumor cells, whereas in situ hybridization methods (namely, chromogenic in situ hybridization (CISH) and fluorescence in situ hybridization (FISH)), are regarded as second-level techniques to be performed in case of equivocal results [16,17]. HER2-positivity is currently defined as intense protein overexpression in >10% of the tumor cells by IHC, or HER2 gene amplification by FISH in breast and gastroesophageal cancer [18]; these classification systems have been applied by most but not all studies, resulting in poor validation.

Using different techniques may yield discordant results, with IHC positivity rates being higher than ISH ones across different studies; further issues include assay standardization and the definition of distinct cut-offs based on the sensitivity level of each method [19]. Though widely observed, especially in NMIBCs, gender differences have not been described in this setting [20]. However, a high concordance between HER2 protein overexpression and gene amplification in BC has been described in studies based on large data sets [18,21,22]. Conversely, other authors have failed to report an optimal correlation between HER2 gene amplification and protein overexpression, possibly due to epigenetic factors and technique-related factors [16,17]. Obviously, literature findings should be weighted in relation to the geographical, clinical, and pathological characteristics, as well as the number of study cohorts.

Over the years, HER2 expression has been evaluated in BC as well, among other potential biomarkers, with a view to implementing its use in clinical practice [23,24]. As a potential therapeutic target, HER2 has been assessed in clinical trials using anti-HER2 monoclonal antibodies and tyrosine-kinase inhibitors, either in monotherapy or combined with conventional chemotherapy, as second-line treatments in patients with advanced or metastatic BC; however, such phase II trials did not yield acceptable results, with low overall response rates [16]. More recently, ADCs targeting HER2 (among other molecules), such as trastuzumab emtansine, trastuzumab duocarmazine, and disitamab vedotin, showed promising results in multi-tumor basket clinical trials; a further such agent with an enhanced pharmacokinetic profile, namely trastuzumab deruxtecan, is currently under evaluation [16].

Herein, we aim to highlight the updated data on the diagnostic, prognostic, and predictive role of HER2 in BC, and provide a critical discussion on current and emerging issues in the field.

## 2. HER2 Expression in Flat and Inverted Urothelial Lesions

Flat urothelial lesions with atypia encompass a spectrum of pathological entities ranging from non-neoplastic to frankly malignant, including reactive atypia, urothelial dysplasia, and carcinoma in situ (CIS) [25]. The differential diagnosis among such lesions in the appropriate clinical framework mostly relies on their appearance in light microscopy [26]; however, even in the context of proper clinical information, the assessment of morphological parameters alone may be not sufficient to distinguish among these lesions, especially between reactive atypia and CIS [25]. Therefore, in the last few decades, selected immunohistochemical markers have been progressively studied and introduced in the routine pathology practice, including HER2. 

In normal urothelium, membranous HER2 expression ranges from lacking to present in the superficial cell layer only and, occasionally, by intermediate cells, with stronger staining on the basal and lateral side [27,28,29,30,31]. This is in keeping with the theory that the orderly maturation of urothelial cells is supported by the coordinated up-regulation and down-regulation of the class I tyrosine kinase receptors, namely HER2, HER3, HER4, and EGFR, respectively [27]. Normal urothelium showing such HER2 expression patterns has been shown to be diploid in FISH analysis [29], accordingly. A few samples of normal or reactive urothelium displayed full-thickness HER2 immunoreactivity in one study, and the staining was described as weak and focal [30].

**Figure 1:**
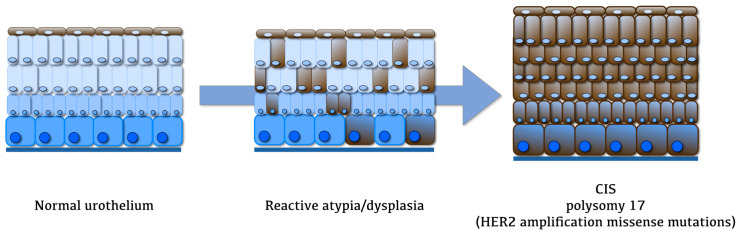
Differential HER2 expression in flat urothelial lesions. CIS: carcinoma in situ.

Wagner et al. [32] described two different HER2 expression patterns in normal, dysplastic, and neoplastic flat urothelium. The diffuse pattern (namely, a focal or diffuse HER2 expression in deeper cell layers) was observed with increasing frequency in mild-to-moderate dysplasia (8/22, 36%) and CIS (12/22, 55%), thus suggesting that HER2 may play a role in malignant transformation. HER2 gene amplification was detected in 33% (2/6) of biopsy samples showing diffuse overexpression of the protein. Conversely, a superficial pattern (namely, HER2 expression in superficial urothelial cells only) was reported more often in patients with mild dysplasia (26/58, 45%) compared to moderate dysplasia (10/58, 17%), normal urothelium in absence of previous BC neoplasms (5/58, 9%), and CIS (3/58, 5%) (Figure 1). 

In keeping with these results, a strong, full-thickness HER2 expression has been described in the majority of CIS cases by several authors [25,28,29,30,31,33]. Two investigators independently evaluated HER2 expression by separately assessing the lower/basal and upper/luminal halves of the urothelium in the study by Gunia et al. [33], yielding significantly different scores in both cases between CIS and non-CIS entities (namely, dysplasia and reactive atypia). Accordingly, Barth et al. analyzed a large panel of luminal and basal markers in a series of 156 CIS cases [31], most of them being characterized by the expression of luminal markers, including HER2; such findings were confirmed in a more recent study from the same group [22]. Interestingly, HER2 expression showed higher specificity in identifying CIS across studies, as compared to other markers (namely, CK20, CK5/6, P53, CD138) [30,33].

An additional advantage in using HER2 as a diagnostic tool is the fact that its positive staining in CIS cases involves the deeper urothelial layers; therefore, HER2 may be applied even to tissue samples whose urothelial lining is not intact, which is a quite common occurrence in TUR specimens [26].

A further point is the higher presence of HER2 immunostaining in morphologically normal urothelium, either from patients with positive as compared to negative BC history (64% versus 33%) in one study [32], or adjacent to urothelial neoplasms [31], thus suggesting that early molecular changes may precede the development of morphologically detectable features of malignancy. In keeping with this, a normal urothelium adjacent to CIS showed weak positivity compared to the moderate to strong expression of HER2 in the series by Barth et al. [31].

HER2 enrichment at the protein level is mostly attributable to polysomy 17 rather than gene amplification in both FISH and SISH analysis [29,31,33,34]. The results from a recent next-generation sequencing (NGS) study on CIS cases reported a rate of missense mutations in the extracellular domain of HER2 as high as 16%, encompassing the pathogenic activating S310F mutation, which is a common HER2 alteration in BC [34].

Beyond its diagnostic role in CIS, HER2 alone or in combination with other agents may be the leading actor of intravesical targeted therapies, including ADCs [33,34], in order to supply an alternative bladder-sparing approach in the subset of BCG-refractory CIS patients otherwise amenable to cystectomy [31,34].

A subset of urothelial lesions, ranging from benign to frankly malignant, displays a partial to diffuse inverted/endophytic pattern of growth; in this scenario, distinguishing lesions with different outcomes may be challenging due to their morphological similarities, hence the need to find diagnostic biomarkers. Since moderate to strong HER2 overexpression has been described in inverted UCs as compared to their benign counterparts both in the bladder and upper urinary tract [35], this might be a useful adjunct in the diagnosis of malignant urothelial lesions with an inverted/endophytic pattern.

## 3. Prognostic Role of HER2 in NMIBC

BC ranks third among all cancers in terms of HER2 overexpression, carrying as much as 6–17% of gene mutations and/or amplification in tissue samples [36].

HER2 protein overexpression has been reported as a marker of poor prognosis in BC. Despite disagreeing results, the finding that the nodal metastases consistently showed HER2 overexpression as compared to the respective primary strongly supports this hypothesis [37,38], although it might be related to tumor heterogeneity. Accordingly, a higher HER2 status was described by several authors as significantly associated with a higher stage and grade and a poor disease-specific survival, mainly in the muscle-invasive and metastatic setting [39,40,41,42,43,44,45]. Furthermore, the higher rates of HER2 positivity in advanced cases may suggest its use as a marker of circulating tumor cells assessed through liquid biopsy, thus overcoming the need to take further tissue biopsies in such patients [46].

The prognostic role of HER2 in NMIBC is more debatable. In a next-generation sequencing analysis of a cohort of 105 NMIBCs of varying stage (pTis, pTa, pT1) and grade (high and low), higher-stage and grade tumors were consistently enriched with HER2 mutations [47]. Increased HER2 expression has been reported in patients with relapsed NMIBC after adjuvant intravesical therapy [48]. HER2 overexpression has been significantly associated with shorter progression-free survival (PFS) [49,50] and especially recurrence-free survival (RFS) [48,51,52], or both [53], whereas earlier studies failed to confirm these findings [54,55,56]. Conversely, HER2-positive tumors from a cohort of 60 NMIBCs had favorable outcomes in terms of lower odds ratios of grade progression at any subsequent biopsy diagnosis [57].

In two further studies, our group reported HER2 overexpression as an independent predictor of RFS and PFS, either alone [58] or in combination with microsatellite instability markers (MLH1 and MSH2) [59], the latter being potential therapeutic targets as well. In our studies, the proposed markers even outperformed BCG treatment in predicting PFS.

Risk stratification tools combining clinical and pathological parameters are currently used to inform patients’ therapeutic and follow-up strategies [3,60,61], despite their less-than-optimal performance, especially in the high-risk group. In this scenario, the available data on the putative prognostic role of HER2 is notably intriguing, since patients with high-risk NMIBCs may benefit from a spectrum of treatment options carrying different side effects and/or risk of failure.

However, in order to best assess the role of HER2 as a risk stratification marker, alone or in combination, some issues need to be fixed. The findings in the literature are biased by a series of inherent limitations that affect the possibility to draw consistent results—mostly (1) the retrospective fashion of available data, (2) intra- and inter-tumor heterogeneity, and (3) discrepancies among assessment methods used in different studies, namely antibody clones, evaluation criteria, and cut-offs adopted for the definition of HER2 positivity [37,45,62,63]. Hence, large multi-center studies are needed in order to overcome such limitations.

## 4. HER2 in Divergent Differentiation and Histological Subtypes of BC

Some types of BCs may show peculiar morphological and biological features, which accounts for them being classified as divergent forms (such as squamous, glandular, trophoblastic) or even distinct subtypes, including micropapillary, plasmacytoid, and sarcomatoid tumors [64]. These variants are believed to carry a worse clinical outcome than conventional urothelial carcinoma (UC), both in the NMIBC and MIBC setting [65], although there is no universal agreement on this point; nevertheless, it is highly recommended to carefully assess these features in tissue samples, even when present to a small extent (see below). The use of reliable prognostic and predictive markers might be of pivotal importance in stratifying such BC patients [66].

Variable rates of HER2 expression have been described in these patients. Behzatoglu et al. reported the presence of HER2 overexpression in 56% of micropapillary carcinomas (MPCs) and only 36% of conventional BCs; on the other hand, HER2 positivity rates declined to 20% in the group of BCs with squamous differentiation (SD-BC), and no expression was seen in the cases of sarcomatoid carcinoma (SCs) and BC with glandular differentiation (GD-BC) [67], in keeping with the findings by Wang et al. on a cohort of upper urinary tract UCs [68]. Even lower positivity rates (3–11%) of HER2 amplification/overexpression in SD-BCs were reported in two studies [69,70].

HER2 alterations have been extensively studied in MPCs [71,72,73], with rates of overexpression and amplification in up to 75% and 42% of cases, respectively [74,75], supporting the classification of MPCs as luminal tumors [7,71]. Two studies comparing stage-matched MPC to conventional BC reported higher amplification rates in the first group (12% and 15% vs. 6% and 9%, respectively) [70,73].

When evaluating the presence of MP architecture in BC, it may happen that such morphological features can be detected only in part of an otherwise conventional UC. There is still no agreement about which proportion of MPC within a tumor yields clinical significance; therefore, any MP component, possibly with its percentage, should be reported [76]. According to Bertz et al., CISH disclosed HER2 gene amplification in 30% (3/10) of the BCs harboring a ≥30% MP component [77]. On the other hand, 77% of the HER2-amplified tumors in the cohort studied by Tschui et al. presented with an MP morphology, ranging from <10% to 100%, and the HER2-positive group had significantly higher rates of morphological heterogeneity than the control group [78]. Interestingly, Isharwal et al. reported frequent intratumoral heterogeneity of HER2 amplification within combined tumors (i.e., containing both MPC and conventional UC), in that the MP component showed higher rates of amplification than the conventional one [79]. Furthermore, the presence of higher HER2 rates in the conventional component in these tumors compared to both pure conventional UC and those not combined with MP tumor [66,79] suggests that HER2 activation may play a role in the carcinogenesis of this variant. Interestingly, a gene-expression meta-cohort study of 2411 tumors hinted at a subclassification of MPCs into HER2-like and mesenchymal-like [80], in keeping with the findings by Han et al. [81].

Since HER2 positivity has been described in conventional BC as well, it cannot be used to support the diagnosis of MPC [82,83]. On the other hand, there is no consensus on how to treat MPC, especially the NMI cases [66]; therefore, the therapeutic implications of HER2 overexpression in this variant are yet to be explored [84]. 

According to some authors, HER2 assessment in MPC may have a prognostic potential, in that some authors have reported an association with worse cancer-specific survival (CSS) after radical cystectomy [73,75]. In keeping with this, HER2 was overexpressed in as many as 70% of patients with angiolymphatic invasion in a cohort of 27 patients analyzed by Goodman et al., both with an early and advanced disease [74].

Moreover, the association between HER2 expression and amplification is not linear [70,84]; Moktefi et al. reported HER2 protein overexpression in as many as 60% of MPC cases, with only 12% showing HER2 amplification with FISH analysis [70]. Such discrepancies may be due to the low frequency of pure MPC, or to other mechanisms supporting the overexpression at the protein level in these tumors, such as mutations in known hotspots, which have been frequently described in MPC [72]. Nevertheless, a rate as high as 40% of activating HER2 mutations has been reported in MPC in the absence of protein overexpression [72]. A further D769N mutation was detected by Tschui et al. in a HER2-amplified tumor, occurring at the same amino acid position than two other mutations (D769H and D769Y) associated with breast cancer, both resulting in the constitutive activation of the enzyme [78].

Plasmacytoid carcinomas (PCs) are rare and biologically aggressive urothelial malignancies, mostly carrying low levels of HER2 expression and amplification [70,85]. Interestingly, a recent study by Kossaï et al. showed that HER2 positivity rates, though overall low in their cohort of PCs (8/32, 25%), were indeed higher as compared to conventional high-grade UCs (0/30) [86].

Small cell neuroendocrine carcinoma (SCNEC) is an uncommon high-grade biologically aggressive malignancy that may affect several organs, including the bladder, carrying poorer outcomes than conventional urothelial BC [64]. A comprehensive whole-genome analysis of these tumors demonstrated a novel in-frame Prt1 oncogene (PVT1)-ERBB2 fusion, resulting in the aberrant expression of the HER2 gene [87,88]. An earlier study reported a 50% positivity of HER2 protein in a cohort of 10 bladder SCNECs [88].

## 5. HER2 and BC Molecular Subtypes

The biological and clinical heterogeneity of BC has led several researchers to develop classification schemes able to mirror this variability and translate it into distinct subtypes according to their mRNA expression profiles in the last decade. The next steps were to combine these findings into a consensus classification and to implement such molecular subtyping as a risk-stratification tool in routine practice, using IHC in order to assess subtype-specific biomarkers at the protein level [7,8].

According to an earlier classification system proposed by the University of Lund, Sweden, MIBCs can be divided in four molecular subtypes by gene expression profiling, namely UroA, UroB, GU, and SCCL tumors [88]. Using IHC, HER2 expression rates were distinctly different among the groups, ranging from strong (GU) to moderate–low (UroA and UroB) to almost absent (SCCL) [89]. Conversely, HER2 overexpression is associated with Clusters I and II and luminal-like tumors, according to the 2014 Cancer Genome Atlas Network (TGCA) and MD Anderson Cancer Center (MDACC) classifications, respectively [90,91]. 

These and other groups reported apparently distinct molecular frameworks including varying numbers of subtypes (from 2 to 5), yet overlapping in the top-level distinction between luminal and basal clusters [11]. Accordingly, the Consensus Molecular Classification of MIBC by the Bladder Cancer Molecular Taxonomy Group, based on the analysis of 1750 transcriptomic profiles from sixteen published datasets and two additional cohorts, identifies six classes, namely luminal papillary (LumP), Luminal Non-Specified (LumNS), Luminal Unstable (LumU), Stroma-rich, Basal/Squamous (Ba/Sq), and Neuroendocrine-like (NE-like), with different biological and clinical/prognostic features [6]. Herein, HER2 amplifications were enriched in the LumU subtype, which features the highest cell cycle activity among luminal tumors (*p* < 0.001), as well as the uppermost somatic mutation load overall (*p* = 0.009) and a poor prognosis [6]. Nevertheless, patients with LumU BCs may show a good response to radiotherapy and ICI (atezolizumab) [6].

According to Kiss et al. [92], the rate of HER2 alterations at both the gene and protein level is higher in the luminal rather than the basal subtype of MIBC. In keeping with this, HER2 has been used as a surrogate marker of luminal phenotype, usually along with GATA3 and CK20, by a few authors [93,94,95]. Accordingly, Yorozu et al. assessed HER2 status (protein overexpression and/or gene amplification) in a series of 148 UCs of the upper urinary tract, reporting that HER2 positivity was significantly associated with the luminal subtype (*p* = 0.0030) and a shorter overall survival at univariate analysis (*p* = 0.0265) [96].

The proposed molecular classifications mostly focus on MIBC. According to the UROMOL study, a comprehensive multi-institutional transcriptional analysis project aiming to classify NMIBC through molecular methods, Class 2 tumors frequently harbored HER2 mutations and were associated to the CIS pathway of progression, carrying higher progression rates to MIBC [97]. 

All in all, the available data support the hypothesis that HER2 may be an optimal candidate marker to be included in a small and effective antibody panel suitable for BC molecular subtyping in clinical practice.

## 6. With a Little Help from AI: A Step beyond on the Way of Standardization

A major issue preventing the implementation of routine biomarker assessment in BC is the poor reproducibility of results due to the subjective evaluation done by light microscopy. In recent years, artificial intelligence (AI)-based techniques in pathology have been steadily implemented, leading to the development of automated image analysis tools with the ability to evaluate whole slide images (WSIs) in order to determine several types of parameters [98,99]. Such digital image analysis (DIA) algorithms quantify the expression of IHC biomarkers in manually outlined region of interests (ROIs) and scan through the whole slide, thus yielding highly reproducible and accurate results [98].

**Figure 2:**
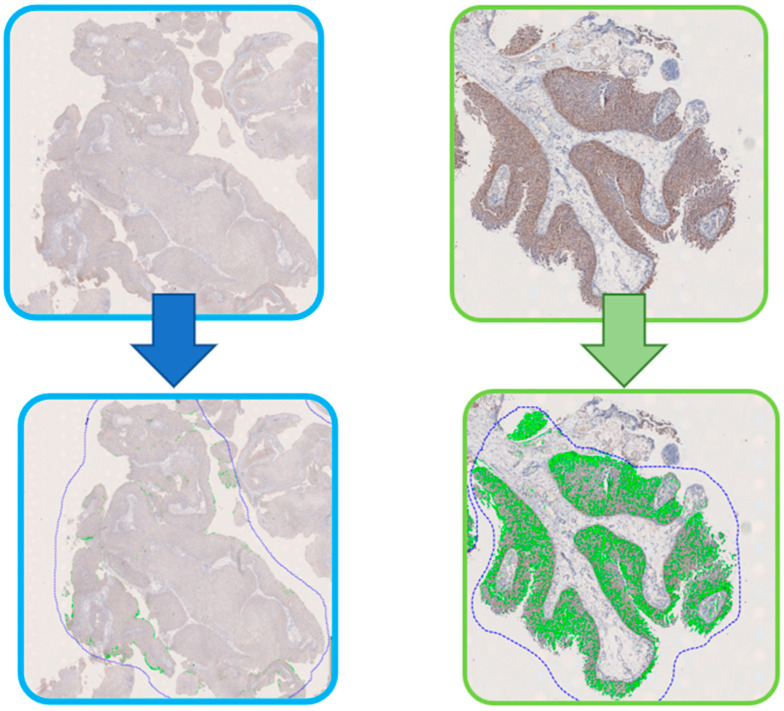
Digital image analysis of HER2-stained BC tissue samples with low (**left**) and high (**right**) protein expression (original magnification 200×).

In routine practice, the semiquantitative assessment of HER2 IHC-stained slides is performed by pathologists manually, as mentioned before, resulting in interobserver variability, in spite of the presence of international widespread guidelines. A further drawback is the relatively high levels of equivocal cases, especially when such evaluation is performed by non-experienced pathologists [18]. DIA may be of pivotal importance in this setting, to the extent that it has been acknowledged as a diagnostic tool for HER2 status evaluation to be implemented into pathology practice according to focused guidelines by the American Society of Clinical Oncology/College of American Pathologists (ASCO/CAP) [100] through several commercially available FDA-cleared and CE-certified algorithms for HER2 IHC quantification [101]. These systems run through a first step of segmentation in order to arrange cells and/or nuclei into discrete staining classes, and they later quantify the percentage of cells in each class. The output may be expressed as membrane connectivity, which is a continual measure of the size distribution of stained membrane fragments ranging from 0 to 1, and is later converted into the guideline-recommended categories of 0, 1+, 2+, 3+ (Figure 2). Since HER2 is a marker of tumor cells only, the step of ROI definition may be skipped; furthermore, sensitivity may be manually set by the user according to perceived staining intensity [98]. 

High rates of agreement between manual and DIA HER2 scoring and between IHC and FISH results have been reported in a study on breast carcinoma [102]. In a recent study, HER2 DIA connectivity showed the strongest association among other prognostic parameters with pathologic complete response in a cohort of HER2+ invasive breast carcinomas treated with anti-HER2 agents in the neoadjuvant setting [103].

The application of such methods in clinical practice needs (1) prior intra-laboratory validation through a comparison with surrogate methods (such as, detection of HER2 at gene level) or consensus images, along with supervision by expert pathologists, and (2) to follow evidence-based guidelines and implementation of regular maintenance and accreditation programs [101,103].

Since FISH is a more complex and time-consuming method than IHC in assessing HER2 status, DIA systems have also been established in order to overcome these issues by automatically detecting, classifying, and counting cells of interest within the slides on the basis of pre-set parameters, including color, intensity, size, pattern, and shape, yielding overall concordance rates approaching 100% [101].

## 7. Conclusions

HER2 is a versatile molecule to be exploited as a diagnostic, prognostic, and predictive tissue biomarker in the assessment of urothelial lesions. Available findings so far suggest that is can be easily implemented in clinical practice, especially with the aid of novel AI-based methods of assessment. In order to define its real potential for patients’ risk stratification and as a therapeutic target, further well-designed and more focused studies are warranted.

## Data Availability

No new data were created or analyzed in this study. Data sharing is not applicable to this article.
